# Cross-cultural validation into Portuguese of a questionnaire to assess computer vision syndrome in workers exposed to digital devices

**DOI:** 10.5935/0004-2749.2022-0256

**Published:** 2023-09-27

**Authors:** Natalia Cantó-Sancho, João Linhares, Elena Ronda-Pérez, Sandra Franco, Esther Perales, Mar Seguí-Crespo

**Affiliations:** 1 Department of Optics, Pharmacology and Anatomy, University of Alicante, 03690 San Vicente del Raspeig, Spain; 2 Physics Center of Minho and Porto Universities (CF-UM-UP), University of Minho, 4710-057 Braga, Portugal; 3 Public Health Research Group, University of Alicante, 03690 San Vicente del Raspeig, Spain; 4 Biomedical Research Networking Center for Epidemiology and Public Health (CIBERESP), 28029 Madrid, Spain

**Keywords:** Computer vision syndrome, Digital devices, Eye health, Validation study, Psychometric properties, Surveys and questionnaires, Síndrome visual do computador, Dispositivos digitais, Saúde ocular, Estudo de validação, Propriedades psicométricas, Inquéritos e questionários

## Abstract

**Purpose:**

As digital devices are increasingly used at work, valid and reliable tools are needed to assess their effect on visual health. This study aimed to translate, cross-culturally adapt, and validate the Computer Vision Syndrome Questionnaire (CVS-Q©) into Portuguese.

**Methods:**

A 5-phase process was followed: direct translation, synthesis of translation, back-translation, consolidation by an expert committee, and pretest. To run the pretest, a cross-sectional pilot study was conducted with 26 participants who completed the prefinal Portuguese version of the CVS-Q© and were asked about difficulties, comprehensibility, and suggestions to improve the questionnaire. To evaluate the reliability and validity of the Portuguese version of the CVS-Q^©^, a cross-sectional validation study was performed in a different sample (280 workers).

**Results:**

In the pretest, 96.2% had no difficulty in completing it, and 84.0% valued it as clear and understandable. CVS-Q© in Portuguese (*Questionário da Síndrome Visual do Computador,* CVS-Q PT©) was then obtained. Validation revealed the scale’s good internal consistency (Cronbach’s alpha=0.793), good temporal stability (intraclass correlation coefficient=0.847; 95% CI 0.764-0.902, kappa=0.839), adequate sensitivity and specificity (78.5% and 70.7%, respectively), good discriminant capacity (area under the curve=0.832; 95% CI 0.784-0.879), and adequate convergent validity with the ocular surface disease index (Spearman correlation coefficient=0.728, p<0.001). The factor analysis provided a single factor accounting for 37.7% of the explained common variance. A worker who scored ≥7 points would have computer vision syndrome.

**Conclusions:**

CVS-Q PT© can be considered an intuitive and easy-to-understand tool with good psychometric properties to measure computer vision syndrome in Portuguese workers exposed to digital devices. This questionnaire will assist in making decisions on preventive measures, interventions, and treatment and comparing exposed populations in different Portuguese-speaking countries.

## Abstract (pt)

**Objetivos:**

À medida que a utilização de equipamentos digitais no emprego aumenta, a avaliação do seu efeito na saúde visual necessita de ferramentas válidas e robustas. Este estudo teve como objetivo traduzir, adaptar culturalmente e validar para português o Questionário da Síndrome Visual do Computador (CVS-Q©).

**Métodos:**

O procedimento foi realizado em 5 fases: tradução direta, síntese da tradução, tradução inversa, consolidação por um painel de especialistas, e pré-teste. Para fazer o pré-teste foi realizado um estudo piloto transversal aplicado a uma amostra de 26 participantes que completaram a versão pré-final da versão portuguesa do CVS-Q©, questionando por dificuldades, compreensão e sugestões de melhoria do questionário. Para avaliar a confiança e validade da versão portuguesa do CVS-Q© foi realizado um estudo transversal de validação em uma amostra diferente (280 funcionários).

**Resultados:**

No préteste, 96.2% dos participantes não apresentaram dificuldades no preenchimento do questionário, enquanto 84.0% indicaram que era claro e compreensível. Obteve-se, então, o CVS-Q© em português (Questionário da Síndrome Visual do Computador, CVS-Q PT©). A sua validação revelou uma boa consistência interna da sua escala (Cronbach’s alpha=0.793), boa estabilidade tem poral (coeficiente de correlação interclasse=0.847; 95% CI 0.764-0.902, kappa=0.839), sensibilidades e especificidades adequadas (78.5% e 70.7%, respetivamente), boa capacidade de discriminação (área abaixo da curva=0.832; 95% CI 0.784-0.879), e uma adequada validade da convergência com o índice de doença da superfície ocular (ocular surface disease index - OSDI; coeficiente de correlação de Spearman=0.728, p<0.001). A análise fatorial revelou um único fator responsável por explicar a variância comum em 37.7%. Um funcionário com uma pontuação ≥7 pontos sofria de síndrome visual do computador.

**Conclusão:**

O CVS-Q PT© pode ser considerada uma ferramenta intuitiva, de fácil interpretação e com boas pro priedades psicométricas para avaliar a síndrome visual do computador em funcionários portugueses expostos a ecrãs digitais. Este questionário facilitará as decisões sobre medidas preventivas, intervenções e tratamento, e a comparação entre as populações expostas em diferentes países de língua portuguesa.

## INTRODUCTION

Computer vision syndrome (CVS), also known as digital eye strain, is a group of visual and ocular symptoms associated with prolonged use of digital devices^(1)^.

Nowadays, the continuing development of new information and communication technologies is likely to increase CVS significantly. In addition, due to the COVID-19 pandemic, remote work has increased in all EU countries; for example, in Portugal, 13.9% of its employers regularly worked remotely in 2020, which is higher than the European average of 12.0%^(2)^. This marked exposure to digital devices is expected to directly affect the population’s visual health, as some authors have reported^(3,4)^.

A recent review of CVS found that the prevalence of CVS-related symptoms ranges from 25.0% to 93.0% in the general population^(1)^. Many studies have shown a high CVS prevalence on computer-using workers from different countries, which generally exceeds 50.0%^(1,5)^. However, the main limitation of most studies lies in using unstructured and unvalidated *ad hoc* questionnaires, which do not guarantee the reliability and validity of the obtained results and makes comparison of results difficult^(1,6)^. To date, only two clinical studies have addressed the prevalence of CVS in the Portuguese population^(7,8)^. The study presented by Dzhodzhua et al.^(7)^ was carried out at a university hospital in Lisbon. They found a CVS prevalence of 92.6%, but the study had a small sample size (n=27) and did not use a specific CVS questionnaire for its diagnosis^(9)^. The Portuguese Group of Ergophthalmology^(8)^ studied digital asthenopia in Portuguese workers to assess the effect of an ergonomic intervention using a specific CVS questionnaire; however, the prevalence value was not presented, and to the best of our knowledge, the psychometric properties of the instrument (reliability and validity) were also not provided.

The Spanish version of the CVS Questionnaire (CVS-Q©) is also available, which was designed and validated to measure CVS as a global construct. It is a self-administered questionnaire that contains 16 items to diagnose CVS and has sensitivity and specificity values >70%, and its test-retest repeatability and psychometric properties are good. It is also intuitive and is easy to understand and apply^(10)^.

Creating a questionnaire from scratch requires money and time investments. Therefore, the translation, cultural adaptation, and validation (TCAV) of tools already designed and of proven quality are recommended options for allowing experiences to be exchanged and comparisons made between different populations and countries, which are extremely necessary in the health field^(11)^. TCAV aims to guarantee the equivalence between the original questionnaire and the adapted version and preserve its psychometric properties^(12,13,14)^.

Therefore, given the growing exposure to digital devices at work, which appears to increase year by year, and given that there is still no validated tool to assess the effect of this situation on workers’ visual health, this study aimed to carry out the TCAV of the original CVS-Q© in Portuguese.

## METHODS

The study was approved by the Ethics Committee of the University of Alicante, Spain (UA-2018-02-22). It was conducted following to the principles of the latest revision of the Declaration of Helsinki. The study consists of two phases: (1) translation and cultural adaptation (TCA), and (2) validation of the Portuguese CVS-Q© version.

### 1. TCA

Based on the original questionnaire (CVS-Q©)^(10)^, TCA was carried out following the phases set out in the scientific literature^(14)^:

1.1. *Direct translation.* Two bilingual (Spanish and Portuguese) translators, whose native language is Portuguese, each completely and independently translated the original questionnaire into Portuguese.

1.2. *Synthesis of translations.* The two translators from the previous phase held a meeting to compare both versions, point out any discrepancies between them, and reach an agreement to obtain the synthesis version in Portuguese.

1.3. *Back-translation.* Two bilingual (Spanish and Portuguese) translators, whose native language is Spanish, independently translated into Spanish the synthesis version obtained in the previous phase.

*1.4. Consolidation by an expert committee.* A multi-disciplinary committee of experts in occupational and visual health, in the TCA of questionnaires, and together with two authors of the original questionnaire and the four translators who participated in the previous phases, was formed. The original questionnaire and each translation obtained in the previous stages were provided to the committee, along with the corresponding reports explaining the reasons for each previously made decision. The whole process was reviewed, and the prefinal questionnaire version in Portuguese was obtained. As the four translators from the first two phases currently live in Spain and may have vocabulary and expression limitations, two external Portuguese collaborators with knowledge of Spanish were asked to perform quality control.

1.5. *Pretest.* In this last phase, the aim was to analyze the TCA quality and the comprehensibility and feasibility of the instrument. A cross-sectional pilot study was performed in a sample of 26 participants (24 adults and 2 adolescents)^(15)^. The participants were recruited by non-probabilistic snowball sampling because of COVID-19 pandemic in May and June 2020. The participants who were Portuguese and lived in Portugal at the time of the study were included. An adaptation of CVS-Q© to an online format was made and, apart from the prefinal Portuguese version, included sociodemographic (sex and age) and exposure (number of hours of using digital devices per day) questions and posttest designed *ad hoc* that comprised both closed and open questions to assess the cognitive debriefing of the prefinal version.

### 2. Validation

A cross-sectional validation study was conducted with Portuguese workers who used digital devices. They were recruited from the University of Minho, Braga, Portugal, between April and December 2021. The inclusion criteria were age 18-65 years and exposure to digital devices on a working day. The exclusion criteria were having undergone refractive or cataract surgery, suffered any ocular pathology during the study, and/or undergoing any ocular treatment (including artificial tears) in the 3 months before the study, which could affect CVS symptomatology.

The sample size necessary to validate an instrument may vary depending on the number of items or dimensions. However, a minimum size of 200 participants is usually recommended to ensure stable results that can be generalized^(16)^. In this study, a final sample of 280 participants was enrolled for questionnaire validation.

Two researchers from the University of Minho were responsible for contacting workers from that university through email. Those interested in participating responded to an adapted Portuguese CVS-Q© version online, and anamnesis included sociodemographic (sex, age, education level, and profession), ocular health (vision-related alterations, pharmacological treatment, and eye surgery), current prescription (habitual optical correction and at work, and its design and related activities), and exposure to digital devices (number of hours using digital devices for work and leisure purposes per day). They also completed an online Portuguese version of the ocular surface disease index (OSDI)^(17)^, a questionnaire that assesses dry eye-related symptoms.

The following psychometric properties were assessed:

2.1. *Reliability.* Internal consistency was evaluated by calculating Cronbach’s alpha coefficient (α) for both global scale and single items, as well as intraobserver reliability (test-retest repeatability), using the intraclass correlation coefficient (ICC), based on a mixed-effects model with a measure of absolute agreement, and using Cohen’s kappa coefficient (κ) to diagnose CVS. To calculate these coefficients, a subsample (n=62) retook the Portuguese CVS-Q© version after 7-15 days. The instrument shows good reliability when these coefficients are >0.7^(18)^.

2.2. *Validity*. Logical and content validity was evaluated by analyzing the pretest questions. To evaluate criterion validity in the absence of a gold standard for CVS diagnosis, the external criterion followed was the same as that used by the authors of the original questionnaire, “appearance of at least one symptom two or three times a week”^(10)^. This criterion was based on a literature review, as in the previous study. Responses to the Portuguese CVS-Q© version were used but differently from the use on finding the usual score. To determine the diagnostic performance of the questionnaire, the sensitivity and specificity of all possible values of the questionnaire total score were calculated, and the area under the curve of the receiver operating characteristic (ROC) was identified to estimate the ability of the scale to diagnose CVS. Finally, factor analysis was performed to determine whether the set of items that constituted CVS-Q PT© had a unidimensional or multidimensional structure. Mardia’s test was performed to assess skewness and kurtosis. Bartlett’s test of sphericity and the Kaiser-Meyer-Olkin (KMO) test were applied to check for the presence of underlying factors. The parallel analysis was conducted to determine the number of factors to retain, and the polychoric matrix was used. A principal component analysis was performed to determine the adequacy of the items to the model and whether any should be removed based on the measure of sampling adequacy (MSA) index. MSA values of <0.50 suggest that the item does not measure the same domain as the remaining items in the pool and should, thus, be removed^(19)^. Subsequently, an exploratory factor analysis, using the robust unweighted least squares method for factor extraction, was run because of its higher power with medium-sized samples. The following robust goodness-of-fit statistics were included to assess the model’s fit: (1) root mean square error of approximation (RMSEA) by taking values of ≤0.10 as an admissible fit; (2) comparative fit index (CFI), for which values of >0.95 are adequate; (3) goodness-of-fit index (GFI), values of >0.95 are indicators of a good model fit; (4) root mean square of residuals (RMSR), using Kelley’s criterion, estimates the reference value to consider an acceptable fit^(20)^; and (5) weighted root mean square residual (WRMR), values of <1.0 represent a good fit^(21,22)^. The following indices were also globally considered to determine dimensionality: (6) unidimensional congruence (UniCo) and (7) mean of item residual absolute loadings (MIREAL). A UniCo value of >0.95 and a MIREAL value of <0.300 suggest that data can be essentially unidimensional^(23)^. If the values of these statistics are within the cut-off points established in the literature, the instrument has adequate construct validity. Likewise, construct validity was evaluated through convergent validity using the OSDI test. Spearman’s correlation coefficient was calculated considering the total score on both questionnaires; and, the Chi-square statistic (χ^2^) was calculated to determine differences between groups with different diagnoses: presence/absence of CVS and dry eye symptomatology. A worker was considered symptomatic when the total score on the OSDI was >13 points^(24)^. The OSDI questionnaire was chosen because it comprises some of the same symptoms as those in CVS but others were very differently related to quality of life or environmental factors. The correlation between the two was expected to be good (as they both assess eye symptoms), but not excellent because they measure different constructs.

Table 1 lists all the indices and statistics calculated to assess the reliability and validity of the instrument, with the correspondent cut-off values to be applied to each methodology.

**Table 1:** Indices, statistics, and coefficients used to assess the reliability and validity of the instrument

Psychometric properties		Name of index, statistic, or coefficient	Abbreviation	Adequate value
**Reliability**	Internal consistency	Cronbach’s alpha coefficient	α	>0.7
	Test-retest repeatability	Intraclass correlation coefficient	ICC	>0.7
		Cohen’s kappa coefficient	κ	>0.7
**Validity**	Redundancy between items	Bartlett’s test of sphericity	-	p<0.001
	Sampling adequacy	Kaiser–Meyer–Olkin	KMO	>0.8
	Adequacy of the items to the model	Measure of sampling adequacy	MSA	>0.5
	Model’s fit	Root mean square error of approximation	RMSEA	≤0.10
		Comparative fit index	CFI	≥0.95
		Goodness-of-fit index	GFI	>0.95
		Root mean square of residuals	RMSR	≈0.06
		Weighted root mean square residual	WRMR	<1.0
	Model’s dimensionality	Unidimensional congruence	UniCo	>0.95
		Mean of item residual absolute loadings	MIREAL	<0.300

### Statistical analysis

For both the TCA (pretest) and validation, a descriptive analysis of categorical variables was performed by calculating their absolute frequency and percentage. For continuous variables, the mean, standard deviation and range were obtained. SPSS Statistics version 28 and FACTOR 10.08.4 were used.

## RESULTS

### 1. TCA

From the direct translation, two Portuguese translations of the original questionnaire were obtained. In the subsequent phase, a single synthesis Portuguese version was obtained. In the back-translation of this synthesis version, two back-translated Spanish CVS-Q© questionnaires were obtained. After the committee meeting and subsequent quality control, a consolidated prefinal questionnaire adapted to Portuguese was obtained (CVS-Q PT©). Table 2 shows the proposals of the expert committee, which were modified during the quality control conducted by the two external collaborators, from which the prefinal version was obtained to be tested in the last TCA phase.

**Table 2:** Decisions from the quality control to obtain the prefinal Portuguese version of the Computer Vision Syndrome Questionnaire based on the consensus of experts

	Quality Control	
Consensus of experts	Proposal from external collaborator	Pre-final version/ Reason or cause
«Questionário da Síndrome da Visão de Computador	«Questionário da Síndrome Visual do Computador»	«Questionário da Síndrome Visual do Computador» Both collaborators agreed to change the title
«A preencher por o trabalhador»	«A preencher pelo trabalhador»	«A preencher pelo trabalhador» Both collaborators agreed to change the term
«Indique se sente algum de estes sintomas, ao longo do tempo usando o computador no trabalho»	«Indique se sente algum dos seguintes sintomas, ao longo do tempo de utilização do computador no trabalho» o «Indique se sente algum destes sintomas, durante o tempo de utilização do computador no trabalho»	«Indique se sente algum dos seguintes sintomas, ao longo do tempo de utilização do computador no trabalho» The second option was selected because it maintained the meaning of the original version
«Em primeiro lugar, a frequência com que aparecem os sintomas, tendo em conta que»	«Em primeiro lugar, a frequência com que aparece o sintoma, tendo em conta que» o «Tenha em conta, em primeiro lugar, a frequência com que aparecem os sintomas»	«Em primeiro lugar, a frequência com que aparece o sintoma, tendo em conta que» This proposal was selected because it maintained the meaning of the original sentence
«REQUENTEMENTE OU SEMPRE= 2 ou 3 vezes por semana ou quase todos os dias»	«FREQUENTEMENTE OU SEMPRE = 2 a 3 vezes por semana ou quase todos os dias»	«FREQUENTEMENTE OU SEMPRE = 2 a 3 vezes por semana ou quase todos os dias» This proposal was selected because it maintained the meaning of the original version
«Lembre-se: si indica NUNCA na frequência, não deve marcar nada em intensidade»	«Lembre-se: se indica NUNCA na frequência, não deve marcar nada em intensidade» o «Lembre-se: se assinalou NUNCA na frequência, não deve assinalar a intensidade»	«Lembre-se: se assinalou NUNCA na frequência, não deve marcar nada em intensidade» A combination between both proposals was done
«Ardor»	«Ardência»	«Ardor» The word “ardor” was not changed because it means the same in both languages
«Comichão»	«Prurido» concepto técnico «Comichão» concepto común	«Comichão» The original proposal was chosen because it is not a technical concept and it can be better understood by the whole population
«Vermelhão ocular»	«Olho vermelho» concepto común «Rubor ocular» concepto técnico	«Olho vermelho» The original proposal was chosen because it is not a technical concept, and it can be better understood by the whole population
«Sensação de peso nas pálpebras»	«Sensação de pálpebras pesadas» o «Pálpebras pesadas»	«Sensação de peso nas pálpebras» The original option was maintained, as the overall meaning did not change
«Dificuldade do focar na visão de perto»	«Dificuldade de focar na visão ao perto» o «Dificuldade ao focar na visão ao perto» o «Dificuldade em focar em visão de perto»	«Dificuldade em focar em visão de perto» This option was chosen as it best expressed the symptom
«Sensação de ver pior»	«Sensação de má visão»	«Sensação de ver pior» The original version was selected in to keep the meaning that was intended to be expressed in the original version
«A preencher por o investigador»	«A preencher pelo investigador»	«A preencher pelo investigador» Both collaborators agreed to change the term
«O resultado de Frequência x Intensidade deve ser recodificado como»	«O resultado de Frequência x Intensidade deve ser registado como»	«O resultado de Frequência x Intensidade deve ser recodificado como» The original sentence was selected as the term “registado” changed the meaning expressed in the original version
«Si a pontuação total é ≥6 pontos, o trabalhador sofre da Síndrome da Visão do Computador»	«Se a pontuação total é ≥6 pontos, o trabalhador sofre da Síndrome Visual do Computador», o «Se a pontuação total é ≥6 pontos, o trabalhador padece da Síndrome Visual do Computador»	«Se a pontuação total é ≥6 pontos, o trabalhador sofre da Síndrome Visual do Computador» Only the term “si” was changed at the beginning of the sentence, as the way of referring to Computer Vision Syndrome

The sample of adults who participated in the pretest (n=24) included 58.3% females. Their mean age was 42.7 ± 15.2 years (mean ± SD), which ranged from 19 to 70 years, and their mean exposure to digital devices was 6.9 ± 3.5 h, which ranged from 1 h to 15 h/day. Two adolescents also participated, a 12-year-old girl and a 13-year-old boy, who used digital devices for 4 and 7 h a day, respectively.

CVS-Q PT© was evaluated by 96.2% of the participants as a questionnaire that is not hard to complete and by 84.0% as a clear and comprehensible questionnaire. No participant indicated having had any difficulty in comprehending a term, and only two of them suggested adding more response options about the intensity of symptoms as an improvement proposal. The adolescents evaluated the questionnaire as simple and did not indicate any improvement proposals. The percentage of the participants who expressed any difficulty or proposal did not reach the necessary 15.0% for any changes to be made^(14)^. Therefore, the final Portuguese CVS-Q© version, named *Questionário da Síndrome Visual do Computador; CVS-Q PT*© (Online Resource 1), was obtained.

### Validation

Of the 343 people who agreed to participate in the validation study, only 280 met the inclusion criteria. Of these participants, 56.1% were females, with a mean age of 45.38 ± 10.24 years within a range between 22 and 65 years. Regarding the current optical correction, 66.1% normally wear glasses. When at work, 62.1% wears glasses with mainly single vision (30.7%) and progressive (23.2%) lenses. Workers use a computer to work an average of 7.11 ± 1.51 h/day, ranging between 4 and 10 h/day, and an average of 9.34 ± 2.03 h/day for digital devices in total (work and leisure), ranging from 4 to 14 h/day (Table 3).

**Table 3:** Sociodemographic characteristics and exposure to digital devices of the validation study sample

	N	%
**Total**	**280**	**100**
Sex		
Female	157	56.1
Male	123	43.9
Age (years)		
≤40	90	32.1
>40	190	67.9
Level of education		
Lower Compulsory Secondary Education	16	5.7
Beyond Compulsory Secondary Education	264	94.3
Profession		
Teaching and research staff	188	67.1
Administration and services staff	92	32.9
Use of optical correction at work		
No	80	28.6
Glasses	174	62.1
Contact lenses	26	9.3
Occupational use of computer (h/day)		
≤6	88	31.4
>6	192	68.6
Total use of digital devices (h/day)		
≤6	19	6.8
6-10	194	69.3
>10	67	23.9

2.1. *Reliability:* A global scale internal consistency of 0.793 was obtained. The range of correlations of each item with the total score was 0.775 for items 1 and 13, “burning” and “increased sensitivity to light”, and 0.790 for item 8, “heavy eyelids”. Good test-retest repeatability was noted for both CVS-Q PT© scores (ICC=0.847; 95% CI 0.764-0.902, p<0.001) and CVS diagnosis (κ=0.839).

*Validity:* Logical and content validity were guaranteed during the TCA, thanks to the participation of the expert committee in the consolidation of the prefinal questionnaire and of users of digital devices (target population) in the pretest, where the majority indicated that the questionnaire did not need to be improved (92.3%) and no one proposed adding/removing any symptoms.

**Figure 1:**
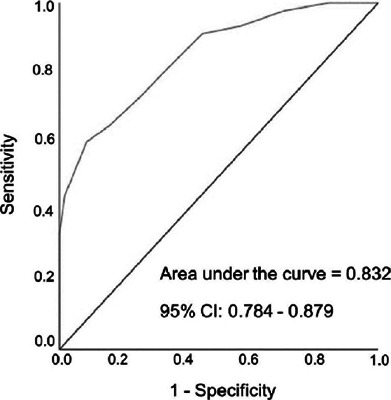
Receiver operating characteristic curve of the Portuguese version of the Computer Vision Syndrome Questionnaire. The area under the curve is 0.832 with 95% CI of 0.784–0.879. This fnding demonstrates the questionnaire’s good discriminant ability.

Regarding the criterion validity, a cut-off point of 7 points would optimize both the questionnaire’s sensitivity and specificity with 78.5% and 70.7%, respectively. Workers who used digital devices and obtained a score ≥7 points in the questionnaire would have CVS. The obtained area under the ROC curve (AUC=0.832; 95% CI 0.784-0.879, p<0.001) indicated the good discriminant ability of the CVS-Q PT© (Figure 1).

Regarding the factor analysis, the results of Mardia’s test indicated that the results were not normally distributed, with skewness of 25.81 (p>0.99) and kurtosis of 293.39 (p=0.03). Bartlett’s statistic result was p<0.001, which suggests a relation between items. A value of 0.74 was obtained in the KMO test, which implies a regular relationship between the items. Therefore, the two assumptions for applying a factor analysis were met. The factor analysis extracted a single factor that accounted for 37.7% of the explained common variance. The MSA values ranged from 0.58 (item 3) to 0.89 (item 13). Thus, no items were dropped from the model. Appropriateness was verified by the robust goodness-of-fit statistics, which gave the following results: RMSEA=0.025, CFI=0.995, GFI=0.974, RMSR=0.065, and WRMR=0.048. A uniCo statistic of 0.942 (95% CI 0.927-0.976) and a MIREAL statistic of 0.273 were obtained (95% CI 0.219-0.305).

Finally, for construct validity, the normality of the two variables to be correlated (CVS-Q PT© score and OSDI score) was verified. Spearman’s correlation coefficient was used, as the variables were not normally distributed. A correlation coefficient of 0.728 was obtained (p<0.001), which implies a good correlation between CVS-Q PT© and OSDI; after performing the Chi-square test, a p<0.001 was obtained, which denotes a significant association between “presence of CVS” and “presence of dry eye-related symptoms” and, thus, demonstrates the construct validity of the instrument. With the validated CVS-Q PT© questionnaire, a CVS rate of 60.0% was noted in the sample of Portuguese workers.

## DISCUSSION

This study resulted in CVS-Q PT©, the Portuguese version of CVS-Q©. This scale was well accepted by the target population and was easy to understand and complete. After its validation, its good psychometric properties for the evaluation and diagnosis of CVS were verified. In this questionnaire, an adult with a score of ≥7 points would have CVS.

When comparing both CVS-Q© versions (original vs. Portuguese), both had similar psychometric properties (Cronbach’s alpha=0.78 vs. 0.79; sensitivity=75% vs. 78.5%; specificity=70.2% vs. 70.7%; AUC=0.826 vs. 0.832; ICC=0.802 vs. 0.847; κ=0.612 vs.0.839)^(10)^. These findings demonstrate that the Portuguese version is reliable and valid for diagnosing CVS as its original counterpart. However, the cut-off point of the Portuguese version is 1 point higher than that in the original questionnaire, but it is the same cut-off point obtained in other linguistic CVS-Q© validations, such as Farsi or Italian^(25,26)^. Slight changes in the cut-off point are common in different linguistic versions of health questionnaires^(27,28)^.

During the validation of this linguistic version, a factor analysis was conducted instead of a Rasch analysis as in the original version, because a Rasch model may be more amenable for the developmental stages of patient-reported outcomes measures^(29)^. As this study aimed to culturally adapt and validate the scale to another language based on an instrument with proven adequate psychometric properties^(10)^, a factor analysis appeared an adequate approach. In this sense, both options are effective for assessing this instrument’s construct validity^(30)^; thus, both Rasch analysis (original and Italian version)^(10,26)^ and factor analysis (in the Portuguese and Farsi version)^(25)^ have been used.

The scientific literature includes two recent articles that mention two different Portuguese questionnaires for CVS assessment^(7,8)^. One is the Portuguese Group of Ergophthalmology Questionnaire, which is a four-item questionnaire with five response options. As it has not been validated, its psychometric properties remain unknown^(8)^. The other is the College of Optometrists in Vision Development Quality of Life-Visual Efficiency Inventory. This is a CVS-nonspecific questionnaire that assesses the effect of visual impairments not only at a visual function level but also on other activities with which vision is closely linked^(7)^. A review attempted to understand the quantitative and qualitative methods currently used to diagnose and evaluate asthenopia in Portuguese air traffic control specialists was also identified^(31)^. This states that very few studies have assessed CVS in the Portuguese population^(31)^ probably because of the lack of a reliable and valid tool to assess this syndrome. Furthermore, as the frequency and intensity of ocular and visual symptoms associated with the intensive use of digital devices are expected to obtain increasingly higher values, especially in the working population, and partly due to the pandemic and remote work^(4)^, a valid and reliable tool like CVS-Q PT© is needed to determine the real effect of digital devices on the visual health of Portuguese workers.

The participation of two of the authors of the original CVS-Q© questionnaire throughout the process is a strong point of this study. In addition, two adolescents aged 12 and 13 participated in the pretest stage because authors have stated that a truly comprehensible questionnaire must be comprehended by a person with knowledge equivalent to that of a schooled individual (aged 10-14 years)^(14,32)^. Regarding limitations, not all the expert committee participants were bilingual (Spanish and Portuguese). An attempt was made to correct this by having two external Portuguese collaborators conducting another quality control. In addition, the original CVS-Q© was designed to be performed in paper format; however, due to the pandemic, the CVS-Q PT© was made using an online support. However, we believe that changing the support in which the questionnaire was presented will not change its psychometric properties.

The CVS-Q PT© has been properly adapted and validated in Portuguese. It will enable further research to estimate the real CVS prevalence in workers exposed to digital devices and identify risk factors in the most susceptible groups. Finally, it will be used by vision professionals to survey the collective visual health of this group. The use of a validated questionnaire in the health surveillance of digital workers is strongly recommended.

## QUESTIONÁRIO DA SÍNDROME VISUAL DO COMPUTADOR

Cross-cultural validation into Portuguese of a questionnaire to assess computer vision syndrome in workers exposed to digital devices

**CVS-Q**

(COMPUTER VISION SVIMDROME QUESTIONNAIRE)

*A preencher pelo trabalhador*

Indique se sente algum dos seguintes sintomas*, ao longo do tempo de utilizaçao do computador no trabalho. Por cada sintoma, marque um X:

a. Em primeiro lugar, a frequência com que aparece o sintoma, tendo em conta que:
  NUNCA = em nenhuma ocasião
  OCASIONALMENTE = de forma esporádica ou uma vez por semana
  FREQUENTEMENTE OU SEMPRE = 2 a 3 vezes por semana ou quase todos os dias
b. Em segundo lugar, a intensidade com que sente o sintoma:
  Lembre-se: se assinalou “NUNCA” na frequência, não deve marcar nada em intensidade.

**Se usa regularmente óculos ou lentes de contacto enquanto trabalha com dispositivos digitais, deve responder pensando em coma se sente enquanto os usa*

**Table:**

	a. Frequência			b. Intensidade	
	NUNCA	OCASIONALMENTE	FREQUENTEMENTE OU SEMPRE	MODERADA	INTENSA
1. Ardor					
2. Comichão					
3, Sensação de corpo estranho					
4. Lacrimejo					
5. Pestanejar excessivamente					
6. Olho vermelho					
7. Dor ocular					
8. Sensação de peso nas pálpebras					
9. Olho seco					
10. Visão turva					
11. Visão dupla					
12. Dificuldade em focar em visão de perto					
13. Aumento de sensibilidade à luz					
14. Halos de cores à volta dos objectos					
15. Sensação de ver pior					
16. Dor de cabeça					

*A preencher pelo investigador*

Cálculo da PONTUAÇÃO TOTAL considerando que:

- Frequência:
  - NUNCA = 0
  - OCASIONALMENTE = 1
  - FREQUENTEMENTE OU SEMPRE = 2
- Intensidade:
  - MODERADA = 1
  - INTENSA = 2
- Gravidade:
  - O resultado de Frequência x Intensidade deve ser recodificado como: 0 = 0; 1 ou 2 = 1; 4 = 2

**Table:**

	Frequência	Intensidade	Frequência x Intensidade	Gravidade
1. Ardor				
2. Comichão				
3. Sensação de corpo estranho				
4. Lacrimejo				
5. Pestanejar excessivamente				
6. Olho vermelho				
7. Dor ocular				
8. Sensação de peso nas pálpebras				
9. Olho seco				
10. Visão turva				
11. Visão dupla				
12. Dificuldade em focar em visão de perto				
13. Aumento de sensibilidade à luz				
14. Halos de cores à volta dos objectos				
15. Sensação de ver pior				
16. Dor de cabeça				

$$\text{Pontuação total}=\sum_{\text{i}=1}^{16}$$

Se a pontuação total è ≥ 7 pontos, o trabalhador sofre da Síndrome Visual do Computador (Computer Vision Syndrome).

## References

[1] Coles-Brennan C, Sulley A, Young G (2019). Management of digital eye strain. Clin Exp Optom.

[2] Eurostat, European Commission (2021). Employed persons working from home as a percentage of the total employment, by sex, age
and professional status.

[3] Ganne P, Najeeb S, Chaitanya G, Sharma A, Krishnappa NC (2021). Digital eye strain epidemic amid COVID-19 pandemic - A cross-sectional
survey. Ophthalmic Epidemiol.

[4] Salinas-Toro D, Cartes C, Segovia C, Alonso MJ, Soberon B, Sepulveda M (2022). High frequency of digital eye strain and dry eye disease in teleworkers during
the coronavirus disease (2019) pandemic. Int J Occup Saf Ergon.

[5] Tauste A, Ronda E, Molina MJ, Seguí M (2016). Effect of contact lens use on computer vision syndrome. Ophthalmic Physiol Opt.

[6] Dessie A, Adane F, Nega A, Wami SD, Chercos DH (2018). Computer vision syndrome and associated factors among computer users in Debre
Tabor town, northwest Ethiopia. J Environ Public Health.

[7] Dzhodzhua V, Serranheira F, Leite ES, Grillo MM, Uva AS (2017). Visual demands and visual fatigue among ophthalmologists. Rev Bras Med Trab.

[8] Vaz FT, Henriques SP, Silva DS, Roque J, Lopes AS, Mota M (2019). Digital Asthenopia: Portuguese Group of Ergophthalmology Survey. Acta Med Port.

[9] Maples WC (2000). Test-retest reliability of the college of optometrists in vision development
quality of life outcomes assessment. Optometry.

[10] Seguí MM, Cabrero-García J, Crespo A, Verdú J, Ronda E (2015). A reliable and valid questionnaire was developed to measure computer vision
syndrome at the workplace. J Clin Epidemiol.

[11] Epstein J, Santo RM, Guillemin F (2015). A review of guidelines for cross-cultural adaptation of questionnaires could not
bring out a consensus. J Clin Epidemiol.

[12] Beaton DE, Bombardier C, Guillemin F, Ferraz MB (2000). Guidelines for the process of cross-cultural adaptation of self-report
measures. Spine.

[13] Gjersing L, Caplehorn JR, Clausen T (2010). Cross-cultural adaptation of research instruments: language, setting, time and
statistical considerations. BMC Med Res Methodol.

[14] Ramada-Rodilla JM, Serra-Pujadas C, Delclós-Clanchet GL (2013). [Cross-cultural adaptation and health questionnaires validation: revision and
methodological recommendations]. Salud Publica Mex.

[15] Sousa VD, Rojjanasrirat W (2011). Translation, adaptation and validation of instruments or scales for use in
cross-cultural health care research: a clear and user-friendly guideline. J Eval Clin Pract.

[16] Ferrando PJ, Anguiano-Carrasco C (2010). Factor analysis as a research technique in psychology. Pap Psicol.

[17] Santo RM, Ribeiro-Ferreira F, Alves MR, Epstein J, Novaes P (2015). Enhancing the cross-cultural adaptation and validation process: linguistic and
psychometric testing of the Brazilian-Portuguese version of a self-report measure for dry
eye. J Clin Epidemiol.

[18] Prinsen CA, Mokkink LB, Bouter LM, Alonso J, Patrick DL, de Vet HC (2018). COSMIN guideline for systematic reviews of patient-reported outcome
measures. Qual Life Res.

[19] Lorenzo-Seva U, Ferrando PJ (2021). MSA: the forgotten index for identifying inappropriate items before computing
exploratory item factor analysis. Methodology (Gött).

[20] Kelley TL (1935). Essential traits of mental life, Harvard studies in education.

[21] Boateng GO, Collins SM, Mbullo P, Wekesa P, Onono M, Neilands TB (2018). A novel household water insecurity scale: procedures and psychometric analysis
among postpartum women in western Kenya. PLoS One.

[22] Lloret-Segura S, Ferreres-Traver A, Hernández-Baeza A, Tomás-Marco I (2014). [Exploratory item factor analysis: A practical guide revised and
updated]. An Psicol.

[23] Ferrando PJ, Lorenzo-Seva U (2018). Assessing the quality and appropriateness of factor solutions and factor score
estimates in exploratory item factor analysis. Educ Psychol Meas.

[24] Wolffsohn JS, Arita R, Chalmers R, Djalilian A, Dogru M, Dumbleton K (2017). TFOS DEWS II diagnostic methodology report. Ocul Surf.

[25] Qolami M, Mirzajani A, Ronda-Pérez E, Cantó-Sancho N, Seguí-Crespo M (2022). Translation, cross-cultural adaptation and validation of the computer vision
syndrome questionnaire into persian (CVS-Q FA©). Int Ophthalmol.

[26] Cantó-Sancho N, Ronda E, Cabrero-García J, Casati S, Carta A, Porru S (2022). Rasch-validated Italian scale for diagnosing digital eye strain: The Computer
Vision Syndrome Questionnaire IT©. Int J Environ Res Public Health.

[27] Miravitlles M, Llor C, Calvo E, Diaz S, Díaz-Cuervo H, Gonzalez-Rojas N (2012). [Validation of the Spanish version of the Chronic Obstructive Pulmonary
Disease-Population Screener (COPD-PS). Its usefulness and that of
FEV_1_/FEV_6_ for the diagnosis of COPD]. Med Clín (Barc).

[28] Doruk C, Çelik M, Kara H, Polat B, Güldiken Y, Orhan KS (2019). Turkish translation and validation of chronic otitis media
questionnaire-12. Turk Arch Otorhinolaryngol.

[29] Cappelleri JC, Jason Lundy J, Hays RD (2014). Overview of classical test theory and item response theory for the quantitative
assessment of items in developing patient-reported outcomes measures. Clin Ther.

[30] Petrillo J, Cano SJ, McLeod LD, Coon CD (2015). Using classical test theory, item response theory, and Rasch measurement theory
to evaluate patient-reported outcome measures: a comparison of worked examples. Value Health.

[31] Passos I, Almada SV, Reis P (2021). Digital-related eye strain in air traffic control specialists: diagnosis and
evaluation in the portuguese air force staff. Rev Bras Oftalmol.

[32] Fukuhara S, Bito S, Green J, Hsiao A, Kurokawa K (1998). Translation, adaptation, and validation of the SF-36 Health Survey for use in
Japan. J Clin Epidemiol.

